# ﻿*Pitcairniaabscondita* (Pitcairnioideae, Bromeliaceae), a hidden novelty from north-western Jalisco, Mexico

**DOI:** 10.3897/phytokeys.189.76464

**Published:** 2022-02-14

**Authors:** Alejandra Flores-Argüelles, Ana Rosa López-Ferrari, Edith González-Rocha, Adolfo Espejo-Serna

**Affiliations:** 1 Departamento de Botánica y Zoología, Centro Universitario de Ciencias Biológicas y Agropecuarias, Universidad de Guadalajara, camino Ing. Ramón Padilla Sánchez 2100, Nextipac, Zapopan, Jalisco, 45200, México Universidad de Guadalajara Zapopan Mexico; 2 Herbario Metropolitano, Departamento de Biología, División de Ciencias Biológicas y de la Salud, Universidad Autónoma Metropolitana Iztapalapa. Apartado Postal 55-535, 09340, Ciudad de México, México Universidad Autónoma Metropolitana Iztapalapa Ciudad de México Mexico; 3 Doctorado en Ciencias Biológicas y de la Salud, Universidad Autónoma Metropolitana, División de Ciencias Biológicas y de la Salud, Ciudad de México, México Universidad Autónoma Metropolitana Ciudad de México Mexico

**Keywords:** Jalisco north coast, *Pitcairnia* subgenus *Pitcairnia*, Costa norte de Jalisco, *Pitcairnia* subgénero *Pitcairnia*

## Abstract

*Pitcairniaabscondita***sp. nov.**, known until now only from the Municipalities of Cabo Corrientes, Mascota, Puerto Vallarta, San Sebastián del Oeste and Talpa de Allende in the State of Jalisco, Mexico, is here described and illustrated. The new taxon was confused with *P.imbricata* for long time, but differs from this species by its green floral bracts with the apex divergent to spreading (vs. red and appressed) and by the appendiculate at the base chartreuse-green petals (vs. not appendiculate yellow petals). Images and a distribution map of the taxa are presented.

## Abstract (es)

Se describe e ilustra *Pitcairniaabscondita***sp. nov**., conocida hasta ahora únicamente de los municipios de Cabo Corrientes, Mascota, Puerto Vallarta, San Sebastián del Oeste y Talpa de Allende, en el estado de Jalisco, México. El nuevo taxon fue por largo tiempo confundido con *P.imbricata*, pero difiere de la misma por sus brácteas florales verdes con el ápice divergente a extendido (vs. brácteas rojas y adpresas) y por sus pétalos verdes chartreuse con apéndices en la base (vs. pétalos amarillos sin apéndices en la base). Se incluyen imágenes y un mapa de distribución de las especies.

## ﻿Introduction

*Pitcairnia* L’Hér. (L’Héritier 1789–1790 [1788]) with ca. 409 spp. (Gouda et al. 2021, continuously updated) is the second richest genus in Bromeliaceae and is mainly distributed in South America, but has an important centre of diversification in Mexico. The only species of the family that grows out of America, *P.feliciana* (A. Chev.) Harms & Mildbraed (Harms and Mildbraed 1938), belongs to this genus. Out of the 19 genera of Bromeliaceae present in Mexico (Espejo-Serna et al. 2004; Espejo-Serna 2012; Espejo-Serna and López-Ferrari 2018, here updated), *Pitcairnia* occupies the third place in number of taxa, with 54 species (including the one described here). In addition, the genus is notable for the number of endemic taxa present in the country, which reaches 47 species (87.03% of the total). For Jalisco, we have so far reported 13 species (Espejo-Serna et al. 2004; Espejo-Serna and López-Ferrari 2018, here updated) and, with this new find, that number increases to 14. Of these, *P.singularis* Flores-Arg., Espejo & López-Ferr., (Flores-Argüelles et al. 2017), *P.lokischmidtiae* Rauh & Barthlott (Rauh and Barthlott 1987; see also Rauh 1987) and the new taxon here proposed are restricted to Jalisco.

During the fieldwork for the fulfilment of the Master’s Thesis of the first author (Flores-Argüelles 2020), we had the opportunity to collect specimens from one species of *Pitcairnia*, which we could not identify. Recently, reviewing specimens of the family Bromeliaceae collected in Jalisco, we found additional material of this species. After a detailed examination of the specimens, we have not been able to assign it to any of the previously-described species of the genus, so we propose it here as new to science.

## ﻿Material and methods

Plants were collected in the years 2019 and 2020 in the Municipality of Cabo Corrientes, Jalisco, Mexico. The gathering of the specimens was carried out in accordance with Lot and Chiang (1986). Measurements and description were made from fresh material and herbarium specimens. The morphological terms used in the description are those proposed by Radford et al. (1974) and Scharf and Gouda (2008). The type material was deposited at Herbario Metropolitano Ramón Riba y Nava Esparza, Universidad Autónoma Metropolitana Iztapalapa (UAMIZ). We revised herbarium material of the genus *Pitcairnia* housed at A, C, CHAP, CICY, ENCB, FCME, GH, HEM, IBUG, IEB, MEXU, MICH, MO, P, SERO, UAMIZ, UC, US, XAL and ZEA (acronyms according to Thiers 2021). To ensure the status of the proposed new species, we revised the protologues, living specimens as well as herbarium specimens and type material of *P.imbricata* (Brongn.) Regel (Regel 1868) and *P.wendlandii* Baker (Baker 1881), the taxa with morphologically most similarities (see Appendix 1). The distribution map of the species was elaborated with the open source geographic information system QGIS (2021), using the data obtained from the herbarium specimen labels.

## ﻿Taxonomic treatment

### ﻿ Pitcairnia abscondita

Taxon classificationPlantaePoalesBromeliaceae

Flores-Arg., López-Ferr., Gonz.-Rocha & Espejo sp. nov.

05DE336E-FC03-562A-8618-4E094ED47FE5

urn:lsid:ipni.org:names:77254836-1

Figs 1 , 2A–B and 3

#### Type.

Mexico. Jalisco: municipio Cabo Corrientes, ejido Las Juntas y Los Veranos, santuario las Guacamayas, 20°25.802'N, 105°18.978'W, 600 m a.s.l., bosque de galería, 25 Jan 2020, flowered in cultivation, 12 Jul 2021, A. Flores-Argüelles, G. Contreras-Félix & J. Novoa 1189 (holotype: UAMIZ in two sheets!).

#### Diagnosis.

Similar to *Pitcairniaimbricata*, but differs by the presence of green, widely oblong to widely ovate floral bracts with the apex divergent to spreading (vs. red elliptic with the apex appressed to the rachis), arcuate corollas (vs. erect), appendiculate, chartreuse-green, 9.4–9.8 cm long, petals (vs. yellow, not appendiculate 6.5–6.6 cm long).

#### Description.

Plant terrestrial or rupicolous, growing frequently along streams, aerial portion of the stem inconspicuous, with underground erect rhizomes ca. 5 cm in diam., flowering 100–110 cm tall. Roots fibrous, thin. Rosettes 40–50 cm high, 90–110 cm diam. Leaves 10 to 50, rosulate, monomorphic, pseudopetiolate; sheaths brown to light brown, with a transverse white band at the base, triangular, 5–6 cm long, 5–6 cm wide at the base, strongly nerved, densely white-tomentose abaxially, entire; pseudopetiole 20–40 cm long, ca. 10 mm wide, involute, margins minutely spinose-serrate, densely white-tomentose abaxially at the base; blades green, linear, attenuate towards the apical portion, 90–170 cm long, 2.5–5.5 cm wide at its widest part, with a central longitudinal channel, entire, very sparsely lepidote adaxially, glabrous abaxially. Inflorescence terminal, simple, erect to arched; peduncle green, cylindrical, 70–77 cm long, 0.7–1.4 cm in diam. at the base; peduncle bracts green, foliaceous, erect, the sheaths appressed, the blades becoming progressively reduced distally, narrowly triangular, 4–35 cm long, ca. 3 cm wide at the base, entire, attenuate to long-attenuate, glabrescent to glabrous on both surfaces; spike terete, 10–50 cm long, 3.5–4 cm in diam., rachis wholly covered by the floral bracts; floral bracts foliaceous, appressed and imbricate, green, widely oblong to widely ovate, 49–52 mm long, 30–32 mm wide, the apex acuminate, divergent to spreading in living plants, the margin hyaline, glabrous on both surfaces, much longer than the sepals. Flowers 25–80 per inflorescence, polystichous, zygomorphic, slightly, but conspicuously arcuate-recurved, sessile, acropetalous; sepals free, green, oblong, 28–30 mm long, 9–11 mm wide, ecarinate, nerved, acute and shortly apiculate, glabrous; petals free, chartreuse green, narrowly oblanceolate, 94–98 mm long, 14–19 mm wide, rounded and very shortly apiculate, with an adaxially basal, oblong, ca. 13 mm long × ca. 6 mm wide, erose appendage, almost completely adnate to the petal; stamens all equal in length, shorter than the petals, filaments whitish, filiform, 71–72 mm long; anthers yellow, linear, 16–17 mm long, basifixed; ovary half superior, greenish-white, ovoid, ca. 15 mm long, ca. 6 mm in diam., glabrous; style linear, arcuate-recurved, ca. 78 mm long; stigma white, conduplicate-spiral (type II sensu Brown and Gilmartin 1984), glabrous. Capsules dark brown when mature, narrowly ovoid in the outline, trigonous in cross section, 19–21 mm long, 6–8 mm in diam., septicidal, glabrous; seeds reddish to light-brown, fusiform, 1–1.5 mm long, long bicaudate, the caudae filiform, ca. 5 mm long each one.

**Figure 1.**
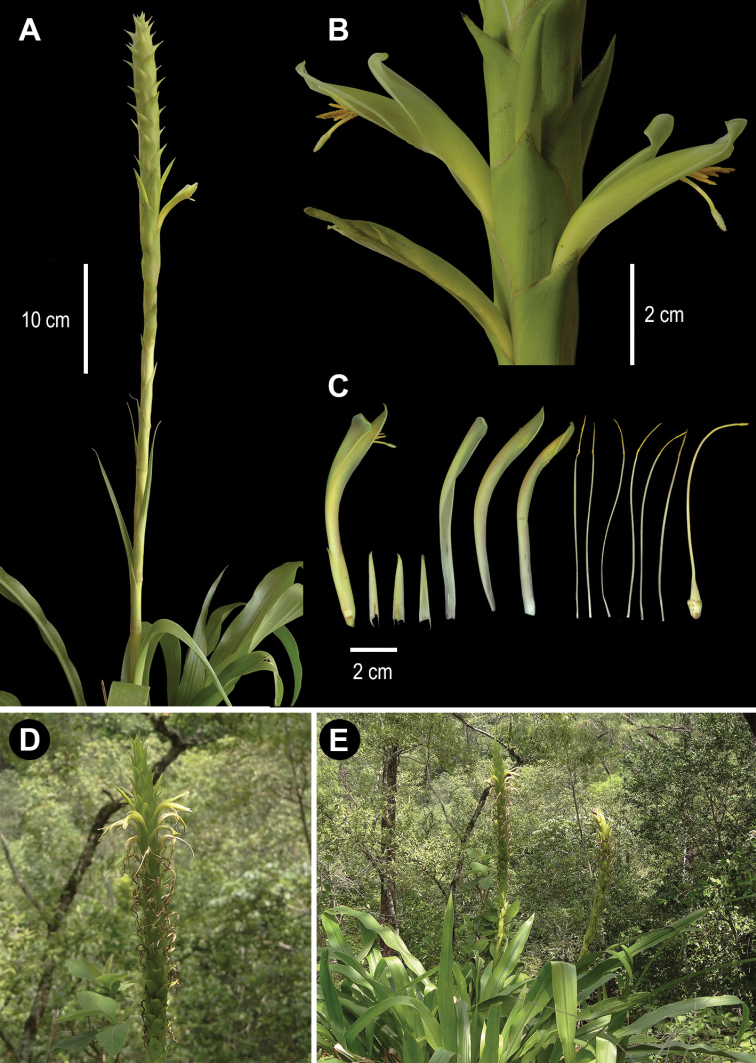
*Pitcairniaabscondita* Flores-Arg., López-Ferr., Gonz.-Rocha & Espejo **A** habit **B** detail of the inflorescence **C** flower dissected **D** inflorescence **E** plant in the type locality (*A. Flores-Argüelles et al. 1131*). Photo credits: A. Espejo-Serna.

#### Etymology.

The specific epithet refers to the fact that, for a long time, the specimens of this species was “hidden” behind the name *Pitcairniaimbricata* (see McVaugh 1989), to difficulties in identifications of herbarium samples, since the dried specimens of *P.abscondita* can be easily confused with *P.imbricata*.

**Figure 2.**
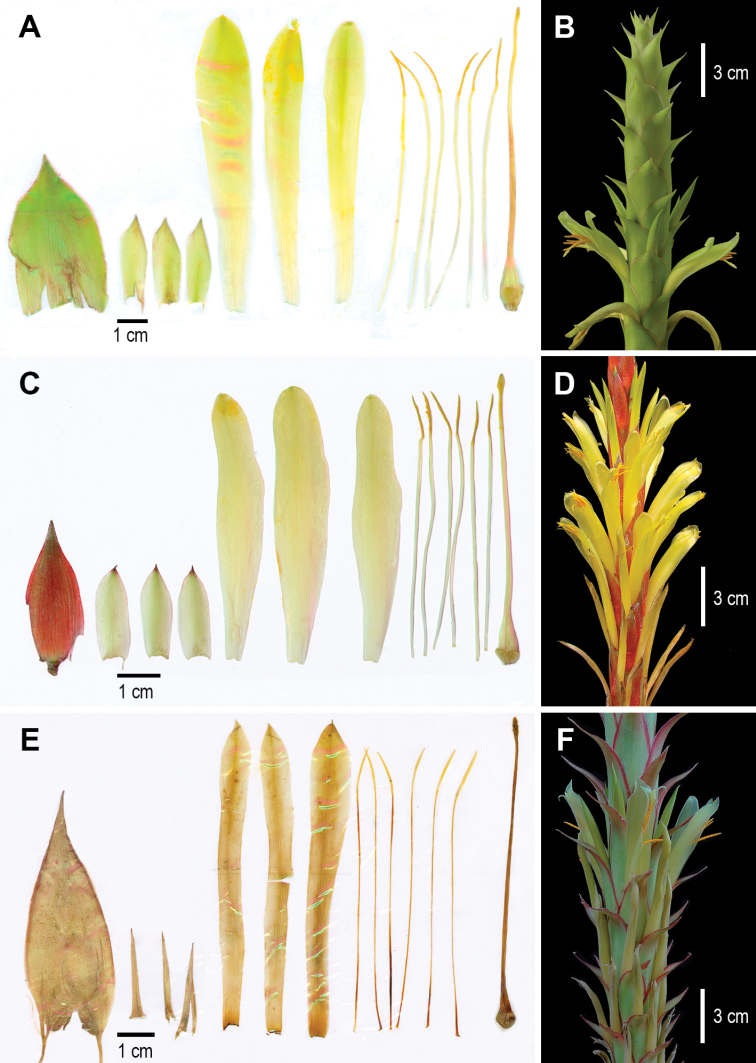
Flower dissected and detail of the inflorescence **A, B***Pitcairniaabscondita* (*A. Flores Argüelles et al. 1189*) **C, D***P.imbricata* (*A. Espejo et al. 7271*) **E, F***P.wendlandii* (*M.I. Mejía-Marín et al. 146*). Photo credits: A. Espejo-Serna.

#### Distribution and habitat.

**Figure 3.**
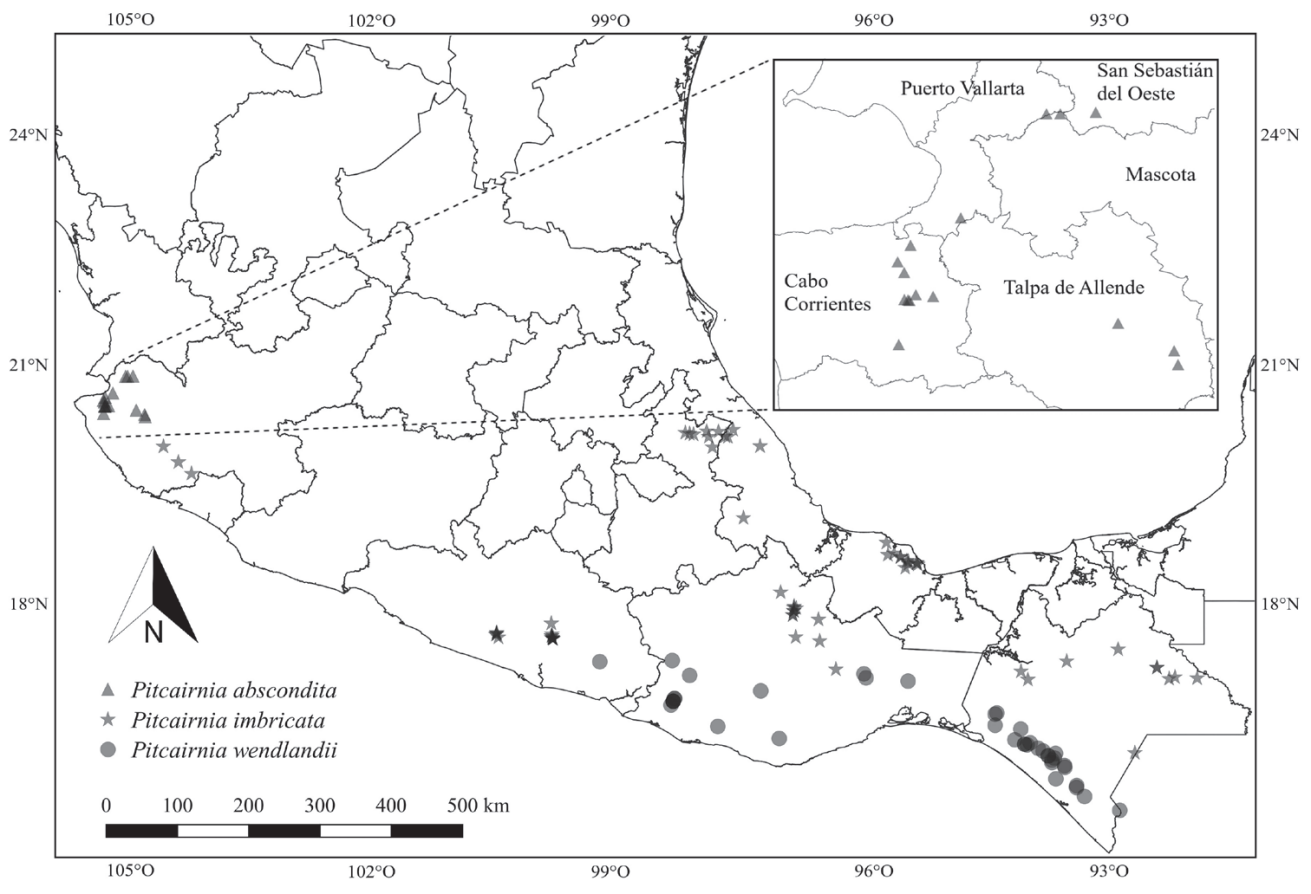
Known distribution of *Pitcairniaabscondita*, *P.imbricata* and *P.wendlandii* in Mexico.

*Pitcairniaabscondita* is known until now only from the State of Jalisco, in the Municipalities of Cabo Corrientes, Mascota, Puerto Vallarta, San Sebastián del Oeste and Talpa de Allende (Fig. 3), growing rupicolous or terrestrial in wet *Pinus*-Quercus forests, gallery forests and cloud forests often near rivers or streams, at an elevation interval from 400–1,500 m a.s.l. Blooms during July and fructifies from August to September.

#### Additional specimens examined (paratypes).

Mexico. Jalisco: Municipio Cabo Corrientes: 3–10 km generally east on the road to Mina del Cuale, from the junction 5 km northwest of El Tuito, 850–1,150 m elev., steep mountainsides pine-oak forest on decomposed granitic soils, with *Podocarpus*, oaks and other deciduous trees in rocky stream valleys, 16–19 Feb 1975, *R. McVaugh 26385* (MEXU (two sheets)); MICH (two sheets)); entre El Tuito y Puerto Vallarta, a 20 km de Puerto Vallarta y a 20 km de El Tuito, ca. 450 m elev., bosque de pino-encino, 19 Jul 1976, *A. Delgado S. & R. Hernández M. 2617* (MEXU); km 18 camino El Tuito hacia la mina de Zimapán, 960 m elev., bosque mesófilo de montaña, 1 Jun 1985, *J.A. Pérez de la Rosa 974* (IBUG); ca. 0.5 km después de Pedro Moreno, rumbo a El Tuito, 637 m elev., 20°24.3833'N, 105°18.2'W, 30 Jul 2003, *J. Ceja*, *A. Espejo*, *A.R. López-Ferrari*, *A. Mendoza R. & I. Ramírez M. 1476* (UAMIZ); km 4 del camino El Tuito-Zimapán, 3.5 km al W de la Provincia, 937 m elev., 20°20.9333'N, 105°17.6067'W, bosque de pino-encino con elementos mesófilos, 2 Aug 2011, *A. Castro-Castro*, *J.G. González*, *R. Guerrero & E. de Castro 2518* (IBUG); km 2 del camino a la mina de Zimapán, 813 m elev., 20°21.0468'N, 105°18.1897'W, bosque de pino-encino, 29 Aug 2019, *A. Flores-Argüelles*, *A.R. López-Ferrari*, *E. González R.*, *J. Hernández B.*, *R. Hernández C. & A. Espejo 1131* (UAMIZ); camino El Tuito-minas de Zimapán, 881 m elev., 20° 21'N, 105°17.7833'W, bosque de pino y encino, 13 Sep 2020, *Brunel*, *E. Ruíz-Sánchez & E. Gándara 901* (IBUG). Municipio Mascota: la Bulera, 9.5 km al WSW de la Estancia, 900 m elev., 20°44.4833'N, 105°0.0333'W, bosque mesófilo de montaña, 2 Apr 2002, *P. Carrillo R.*, *E.M. Barba & M. Alcázar 3147* (IBUG, UAMIZ). Municipio Puerto Vallarta: 800 m de Peña Blanca, camino a Talpa de Allende, 1,360 m elev., 20°44.4767'N, 105°01.6467'W, bosque mesófilo de montaña con *Magnolia*, *Clusia*, *Calophyllum*, *Chamaedorea*, *Chryosophila*, *Cecropia*, *Quercus*, 22 Feb 1998, *R. Ramírez D.*, *F. Cupul*, *H. Hernández*, *J. Fonseca & F. Rodríguez Z. 5252* (IBUG); Ojo de Agua, 6 km al SE de Vallejo, 1,190 m elev., 20°31.337'N, 105°11.6212'W, bosque de encino con *Quercusmagnoliifolia*, *Braheasarukhanii*, *Bejariamexicana*, 21 Jan 2013, *A. Flores-Argüelles & R. Romero 652* (IBUG, ZEA). Municipio San Sebastián del Oeste: los Ojos de Agua, ca. 3.7 km en línea recta al SW de la Estancia de los Landeros, 1,280 m elev., 20°44.665'N, 104°55.9017'W, bosque de galería con *Podocarpus*, *Hedyosmum*, *Saurauia*, 24 Jul 2014, *P. Carrillo R.*, *D. Cabrera-Toledo*, *L.A. Ortega-Valencia & L.M. Valadez-Sandoval 7439* (IBUG). Municipio Talpa de Allende: steep mountains 11–12 miles south of Talpa de Allende, in the headwaters of a west branch of Río de Talpa, 1,200 m elev., 20°14.5833'N, 104°46.7757'W, barranca above a rapid clear stream, in dense Forest of *Quercus*, *Carpinus*, *Distylium*, *Magnolia*, *Podocarpus*, with pine forest on the ridges above, 18–19 Oct 1960, *R. McVaugh 20441* (MICH); brecha Talpa-La Cuesta, 1,400 m elev., bosque mesófilo de montaña, 29 Jul 1990, *R. Ramírez D. & R.G. Tamayo 2139* (IBUG); 15 km al SW de Talpa, 5 km de Aranjuez, 2 km al NNE de la estación de microondas, 1,320 m elev., 20°18.05'N, 104°53.3’W, bosque de pino-encino con asociación de *Pinusdouglasiana*, *P.jaliscana*, *P.oocarpa*, *Quercusmagnoliifolia*, *Q.elliptica*, 16 Jul 2009, *L.M. González-Villarreal*, *J.A. Pérez de la Rosa & G. Vargas 5180* (IBUG).

#### Comments.

In herbarium specimens, *Pitcairniaabscondita* superficially resembles *P.imbricata* and/or *P.wendlandii*. However, the new taxon differs from these two species by the characters shown in Table 1 and Fig. 2. In fact, the Jalisco material that McVaugh (1989) included in his Flora Novo-Galiciana as *P.imbricata*, actually belongs to the new species.

**Table 1.** Comparative features of *Pitcairniaabscondita* with *P.imbricata* and *P.wendlandii* (see also Figs 1 and 2).

	***P.abscondita***	***P.imbricata***	***P.wendlandii***
Leaf blades (cm)	90–170 × 4.5–5.5	70–120 × 5.5–6	50–110 × 4–4.5
Floral Bracts (mm)	widely oblong to widely ovate, green, divergent to spreading at the apex; 49–52 × 30–32	elliptic, red, appressed at the apex; 35–57 × 13–24	elliptic, red to greenish-red, divergent to spreading at the apex; 65–66 × 22–25
Flowers	arcuate-recurved	straight	straight
Sepals (mm)	oblong, acute apiculate; 28–30 × 9–11	oblong apiculate; 21–22 × ca. 8	narrowly triangular, acute; 20–21 × ca. 3
Petals (mm)	narrowly oblanceolate, chartreuse green; 94–98 × 14–19	narrowly oblong, yellow; 65–66 × 12–13	narrowly oblong, to linear, yellow-greenish; 69–70 × 9–10
Anthers (mm)	16–17	ca. 11	13–14
Distribution (Mexico)	Jalisco	Chiapas, Guerrero, Jalisco, Oaxaca, Puebla, Veracruz	Chiapas, Guerrero, Oaxaca

The flowers of *Pitcairniaabscondita* last only one night, opening between 7.30 and 9.30 pm and remain that way during the night, starting to close at 7.30 in the morning, being completely closed at 9.00 am, so they are likely associated with a pollination syndrome by moths or bats, different from *P.imbricata* which, due to its red floral bracts and yellow flowers of diurnal anthesis, is associated with an ornithophilic pollination syndrome (Proctor et al. 1996).

As far as we know, the plants of *P.abscondita* are not used by the inhabitants of the region, so we think that the species has no immediate human pressure; however and due to the lack of detailed information about the precise distribution of the species, we suggest the inclusion of the new taxon in the Not Evaluated (NE) category of the IUCN (2020).

## Supplementary Material

XML Treatment for Pitcairnia abscondita

## ﻿Appendix 1

Specimens examined.

**1. *Pitcairniaimbricata* (Brongn.) Regel**

Mexico. Chiapas: Municipio Berriozábal, *C.R. Beutelspacher 29652* (HEM), *D.E. Breedlove & R.F. Thorne 30896* (MEXU); municipio de Chilón, *D.E. Breedlove 34566* (ENCB; municipio El Bosque, *D.E. Breedlove & R.L. Dressler 29831* (MEXU, MO); municipio La Trinitaria, *D.E. Breedlove 56560* (ENCB); municipio Ocosingo, *E. Martínez S. 17047* (CICY, MEXU), *E. Martínez S. 17610* (MEXU, MO), *E. Martínez S. et al. M-21912* (MEXU), *G. Aguilar M.* et al. *6924* (UAMIZ), *P.E. Valdivia Q. 2418* (XAL). Guerrero: Municipio Atoyac de Álvarez, *C.A. Granados* et al. *370* (MEXU), *J.C. Soto N. & F. Solórzano G. 12815* (MEXU, UAMIZ), *P. Tenorio L.* et al. *487* (MEXU, MO), *V.C. Aguilar J. 642* (FCME), *Y. Ramírez-Amezcua et al. 905* (IEB); municipio Chilpancingo de los Bravo, *E. Matuda 38683* (ENCB, FCME, MEXU), *E. Matuda & colaboradores 38711* (MEXU), *H.E. Moore Jr. 5112* (GH, UC, US), *H.E. Moore Jr. 8130* (MEXU, US), *H.E. Moore Jr. & C.E. Wood Jr. 4705* (A, MICH, US), *H. Kruse 798* (FCME, MEXU), *J. Ceja et al. 1678* (IEB, UAMIZ), *L.A. Kenoyer C246* (GH), *R.M. Fonseca 1725* (FCME); municipio Mochitlán, *G. Espinosa F. 316* (FCME). Jalisco: Municipio Casimiro Castillo, *R. Cuevas G. et al. 6547* (ZEA); municipio Cuautitlán de García Barragán, *A. Vázquez & R. Zúñiga 4457* (ZEA); municipio Villa de Purificación, *G. Morales et al. 116* (IBUG, ZEA), *J.L. Rodríguez et al. 297* (ZEA), *L. Guzmán H. et al. 5239* (ZEA). Oaxaca: Municipio San Felipe Usila, *G. Ibarra M. et al. 3748* (MEXU, MO); municipio San Juan Bautista Valle Nacional, *A.R. López-Ferrari et al. 3196* (UAMIZ), *R. Torres C. & L. Cortés A. 7250* (MEXU); municipio San Juan Juquila Vijanos, *X. Munn et al. 8* (MEXU); municipio San Miguel Quetzaltepec, *B. Rendón A. et al. 1391* (UAMIZ); municipio Santiago Comaltepec, *A. Mendoza R. et al. 280* (UAMIZ), *A. R. López-Ferrari et al. 2117* (UAMIZ), *J. Santana & L. Pacheco 913* (UAMIZ), *J. Utley & K. Burt-Utley 6739* (MEXU), *W.L. Graham 1414* (MICH); municipio Santiago Jocotepec, *B.P. Reko 4128* (US); municipio Totontepec Villa de Morelos, *R. Torres C. & L. Cortés A. 10441* (MEXU). Puebla: Municipio Chignautla, *F. Liebmann 7953* (C, MO); municipio Cuetzalan del Progreso, *J.L. Contreras J. 6134* (CHAP); municipio Hueytamalco, *W. López-Forment s. n.* (MEXU); municipio Huitzilan de Serdán, *G. Toriz A. et al. 621* (CHAP, MEXU); municipio Tepango de Rodríguez, *J. García-Cruz et al. 1413* (UAMIZ); municipio Xochitlán de Vicente Suárez, *I.N. Gomez-Escamilla & B.E. Téllez-Baños 190* (UAMIZ); municipio Yaonáhuac, *P. Tenorio L. et al. 14066* (MEXU). Veracruz: Municipio Atzalan, *F. Ventura 17297* (ENCB, IEB, MEXU, XAL); municipio Catemaco, *A. Gómez-Pompa 5148* (XAL), *S. Sinaca 44* (MEXU), *S. Sinaca 176* (MEXU, MO), *W. Boege 3183* (MEXU): municipio Fortín, *E. Bourgeau 1778* (MO, P); municipio Pajapan, *J.I. Calzada 10899* (XAL), *M. Nee & J.I. Calzada 22725* (GH, XAL), *M. Nee et al. 24975* (GH); municipio San Andrés Tuxtla, *S. Sinaca C. et al. 989* (MEXU); municipio Soteapan, *J.I. Calzada et al. 11439* (XAL), *M. Cházaro & P. Sánchez 3545* (XAL), *T.P. Ramamoorthy et al. 3891* (CICY, MEXU); municipio Tlapacoyan, *F. Miranda 3305* (MEXU); *F. Ventura 13131* (ENCB, IEB); municipio Yecuatla, *C. Gutiérrez B. & M. Cházaro B. 1572* (XAL).

**2. *Pitcairniawendlandii* Baker**

Mexico. Chiapas: Municipio Acacoyagua, *E. Matuda 17729* (MEXU, MO), *N. Martínez-Meléndez 916* (HEM); municipio Ángel Albino Corzo, *M.A. Pérez Farrera 1166* (HEM); municipio Jiquipilas, *D.E. Breedlove 23970* (ENCB), *M.A. Pérez Farrera 463* (HEM); municipio La Concordia, *D.E. Breedlove 40123* (MEXU), *G. del C. López H.159* (HEM), *J. Martínez-Meléndez 641* (UAMIZ), *J. Martínez-Meléndez 1344* (HEM), *N. Martínez-Meléndez 224* (HEM), *R.J. Hampshire & A. Reyes-García 1260* (MEXU), *R. Martínez-Camilo 789* (HEM); municipio Mapastepec, *J.I. Calzada et al. 9006* (MEXU, XAL), *M. Heath & A. Long 1166* (MEXU); municipio Tonalá, *M.A. Pérez-Farrera 425* (HEM); municipio Unión Juárez, *E. Martínez S. & A. Reyes 20310* (IEB, MEXU, MO); municipio Villa Comaltitlán, *C.R. Beutelspacher 22799* (HEM); municipio Villa Corzo, *F. Hernández-Najarro 792* (HEM), *H. Gómez D. 623* (HEM), *J. Martínez-Meléndez 462* (HEM), *J. Martínez-Meléndez 490* (HEM), *J. Martínez-Meléndez 1618* (HEM), *M.A. Pérez Farrera 488* (HEM), *M.A. Pérez Farrera 709* (HEM). Guerrero: Municipio San Luis Acatlán, *F. Lorea 4884* (FCME). Oaxaca: Municipio Asunción Ixtaltepec, *R. Torres C. & C. Martínez R. 6087* (MEXU, MO); municipio Guevea de Humboldt, *R. Torres C. & C. Martínez R. 5948* (MO); municipio Putla, *E. Solano C. & J.C. Gutiérrez H. 4244* (UAMIZ); municipio San Juan Colorado, *M.I. Mejía Marín et al. 146* (UAMIZ), *M.I. Mejía-Marín et al. 325* (UAMIZ), *M.I. Mejía-Marín et al. 704* (UAMIZ), *M.I. Mejía-Marín et al. 720* (UAMIZ), *M.I. Mejía-Marín et al. 802* (UAMIZ), *M.I. Mejía-Marín et al. 900* (UAMIZ), *M.I. Mejía-Marín et al. 928* (UAMIZ); municipio San Mateo Río Hondo, *A. García-Mendoza & F. Martínez 2687* (MEXU, MO); municipio Santa Catarina Juquila, *A. Espejo et al. 7271* (UAMIZ); municipio Santa Cruz Itundujia, *A. Nava Z. et al. 2027* (SERO), *K. Velasco G. et al. 2144* (SERO); municipio Santiago Lachiguiri, *sin colector s. n*. (MEXU).
